# Addendum: Mitochondrial somatic mutation and selection throughout ageing

**DOI:** 10.1038/s41559-025-02640-8

**Published:** 2025-01-30

**Authors:** Isabel M. Serrano, Peter H. Sudmant

**Affiliations:** 1 Center for Computational Biology, University of California, Berkeley, CA USA; 2 Department of Integrative Biology, University of California, Berkeley, CA USA

**Keywords:** Evolutionary biology, Evolutionary genetics

Addendum to: *Nature Ecology & Evolution* 10.1038/s41559-024-02338-3, published online 15 February 2024.

In our original manuscript, we investigated whether there existed a preference for alleles that re-aligned nuclear and mitochondrial ancestry. To address this question, we used mouse strains that were bred such that they carried C57BL/6J nuclear ancestry and differed at mitochondrial haplotypes (original Fig. 1b). We concluded that haplotype sites showed a higher mutation frequency than all other sites in the mitochondrial genome, and that the B6 allele was the predominant alternative allele at these sites (original Fig. 6 and last sentence in abstract). Moreover, for strains that differed from B6 mitochondrial ancestry at 1–3 sites, the frequency of the B6 allele increased with age. Shortly after publication, reviewer Dr. Konstantin Khrapko brought to our attention that this signal may have been due to low-level sample contamination. In our original analyses, we tested and corrected for potential cross-sample and nuclear-embedded mitochondrial DNA sequence (NUMT) contamination (original Supplementary Figs. 12 and 16). We excluded samples that showed signals of contamination (original Supplementary Fig. 12) from our study; however, these analyses may not explicitly account for very low-level contamination between samples. Identifying this level of contamination is challenging because it is difficult to distinguish true low-frequency mutations at haplotype sites from erroneous calls that may be due to low-level contamination. Because our analysis of reversion mutations (original Fig. 6) enriches for B6 allele calls, we applied a correction factor (refer to original Supplementary Fig. 16 and Methods section ‘Estimation of and correction for NUMT contamination’). Our original analyses assumed that the chromosome 1 NUMT would be the major source of likely contamination. Upon re-examination of our data, we find that this correction factor was a highly conservative estimate of overall cross-sample contamination as well (original Supplementary Fig. 16 and Fig. 1). Thus, in principle our analyses should have already corrected for potential low-level contamination across chromosome M at 6394–11041. Importantly, this region contains 9 of the 13 cases that we proposed exhibited significant age-associated increases in reversions (original Fig. 6c). However, we do wish to highlight that very low-level contamination could contribute to the remaining signal of reversion mutations. We report our reanalysis below.

**Fig. 1:**
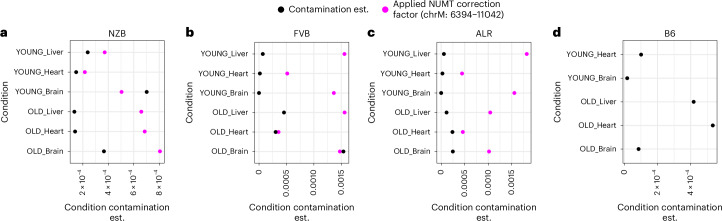
Comparison of conservative contamination estimates and applied chr1 NUMT correction factors. Contamination estimates (black) are shown for each biological condition alongside the correction factor that was applied (pink). Although this correction factor was applied to account for potential NUMT contamination, it serves as a conservative estimate of overall intersample contamination as well.

To estimate potential low-level, cross-sample contamination, we used two methods. First, we examined NZB samples at clusters of NZB haplotype sites (i.e., regions that had haplotype sites <130 bp from each other). Mutations found in multiple nearby sites in such clusters that match the B6/ALR/FVB haplotype are likely to be contamination. Importantly, we selected sites that were found in all B6/ALR/FVB haplotypes but not NZB, thus quantifying total contamination from all other samples into NZB (as previously shown in original Supplementary Fig. 16). Within each cluster, we quantified the contamination frequency as the minimum B6/ALR/FVB frequency among all sites, under the assumption that contaminating reads are those with mutations across all clustered sites. We then took the maximum contamination frequency across clusters as a highly conservative estimate of the total contamination (Fig. 1a). In total, we estimate that NZB samples exhibit contamination levels ranging from 0 to 7 × 10^–4^. Second, we estimated contamination levels across FVB, ALR and B6 samples by examining all haplotype sites. We used the highly conservative assumption that any mismatched conplastic allele found in these samples is derived from contamination. For example, FVB alleles at the FVB haplotype site found in ALR samples are assumed to be evidence of contamination. We summed all independent measurements of contamination stemming from different sources to estimate a total maximum possible contamination (i.e., summing contamination from all other strains). Using this approach, in total we estimate contamination levels ranging from 0 to 1.5 × 10^–3^ across different biological conditions (Fig. 1). Comparing our conservative estimates of contamination to our previously applied correction factor, we find that in all cases but two, the estimated contamination from other samples falls below our already implemented correction factor (Fig. 1). Nevertheless, the presence of even extremely low levels of contamination may well confound analyses of reversion mutations.

We additionally estimated how potential contamination might impact our other analyses by quantifying how our estimates of mutation frequency could be impacted by low-level contamination. The error in the mutation frequency in a sample (${f}_{{\rm{s}}}$) due to contamination can be estimated as the difference between ${f}_{{\rm{s}}}$ and the product of the mutation frequency in the contaminating sample (${f}_{{\rm{c}}}$) and the level of contamination/mixing (${m}_{{\rm{c}}}$) (i.e., ${f}_{{\rm{s}}}-{f}_{{\rm{c}}}\times {m}_{{\rm{c}}}$*)*. For simplicity, we assume that ${f}_{{\rm{s}}}={f}_{{\rm{c}}}$. From these analyses, we estimate an error rate that is three to four orders of magnitude lower than our estimates of the mutation frequency (Fig. 2) in our analyses, thus having minimal impact.

**Fig. 2:**
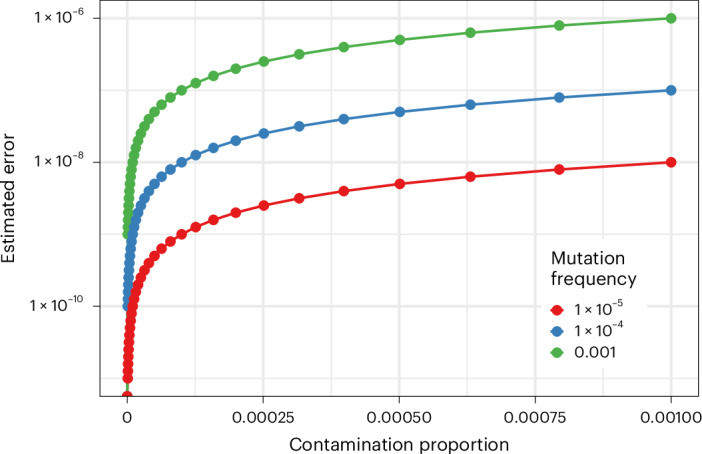
Estimated rate of error in mutation frequency estimates for differing levels of contamination. The estimated error in mutation frequency is plotted as a function of the contamination proportion for sites with different mutation frequencies. In all cases the error rate is several orders of magnitude less than the true mutation frequency.

Overall, we conclude that the correction factor we implemented captures low-level contamination in 9 of the 13 cases that we proposed exhibited significant age-associated increases in reversions. However, we highlight that such low-level contamination could contribute to signals of reversion mutations. We also determine that such contamination would not impact other analyses.

