# Author Correction: Mitochondrial somatic mutation and selection throughout ageing

**DOI:** 10.1038/s41559-025-02641-7

**Published:** 2025-01-30

**Authors:** Isabel M. Serrano, Misa Hirose, Charles C. Valentine, Sharon Roesner, Elizabeth Schmidt, Gabriel Pratt, Lindsey Williams, Jesse Salk, Saleh Ibrahim, Peter H. Sudmant

**Affiliations:** 1 Center for Computational Biology, University of California, Berkeley, CA USA; 2 Lübeck Institute of Experimental Dermatology, University of Lübeck, Lübeck, Germany; 3 TwinStrand Biosciences, Seattle, WA USA; 4 College of Medicine, Khalifa University, Abu Dhabi, UAE; 5 Department of Integrative Biology, University of California, Berkeley, CA USA

**Keywords:** Comparative genomics, Genetic variation

Correction to: *Nature Ecology & Evolution* 10.1038/s41559-024-02338-3, published online 15 February 2024.

The title of this paper has been changed from “Mitochondrial haplotype and mito-nuclear matching drive somatic mutation and selection throughout ageing” in light of the potential impact of low-level contamination on some of the results of this study, which is presented in the Addendum.

