# Supplementary material for: Mitochondrial haplotype and mito-nuclear matching drive somatic mutation and selection throughout ageing
Source: Nat Ecol Evol. Author manuscript; Available in PMC 2024 May 21. (PMC11090800; doi:10.1038/s41559-024-02338-3)
Supplement: Supplementary Materials [file NIHMS1994496-supplement-Supplementary_Materials.pdf]

nature ecology & evolution

Article https://doi.org/10.1038/s41559-024-02338-3

Mitochondrial haplotype and mito-nuclear matching drive somatic mutation and selection throughout ageing

In the format provided by the authors and unedited

Supplemental Materials

Supplementary Note 1: Input parameters for the duplex sequencing pipeline configuration file

A configuration file was created to process samples in each experimental group. The following python script was used to generate the configuration file. The file is also available at https://github.com/sudmantlab/conplastic_mt_profiling.

```
import pandas as pd
sample_names = pd.read_csv("conplastic_sample_names", sep = "\t", header
= None)
sample_names.rename(columns = {0: "sample"}, inplace = True)

df = pd.DataFrame()
df["sample"] = sample_names["sample"]
df["rglb"] = sample_names["sample"]
df["rgpl"] = sample_names["sample"]
df["rgpu"] = sample_names["sample"]
df["rgsm"] = sample_names["sample"]

#hardcoded but will remain the same regardless of the directory created
-- will change if reference and bedfile
#paths change or if a different organism is used
df["reference"] =
"/global/scratch/isabel_serrano/Sudmant_Lab/mm10_chr1NUMT_masked/mm10_NU
MT_masked.fa"
df["target_bed"] =
"/global/scratch/isabel_serrano/Sudmant_Lab/mm10_chr1NUMT_masked/mm10.be
d"
df["blast_db"] = "."
df["targetTaxonId"] = "10090"
df["baseDir"] = "duplex_seq_data"
df["inbam"] = df["sample"] + ".bam"
df["mqFilt"] = 0
df["minMem"] = 3
df["maxMem"] = 200
df["cutOff"] = 0.7
df["nCutOff"] = 1
df["umiLen"] = 18
df["spacerLen"] = 0
df["locLen"] = 10
df["readLen"] = 151
df["clipBegin"] = 10
df["clipEnd"] = 0
df["minClonal"] = 0
df["maxClonal"] = 0.1
```

```
df["minDepth"] = 100
df["maxNs"] = 1
df["runSSCS"] = "FALSE"
df["recovery"] = "noRecovery_noSynLink.sh"
df.to_csv("config.csv", header=True, index=False, sep = ",")
```

Supplementary Figures

Fig S1: Pilot study informed trimming 5’ end of read by 10 bp in this study

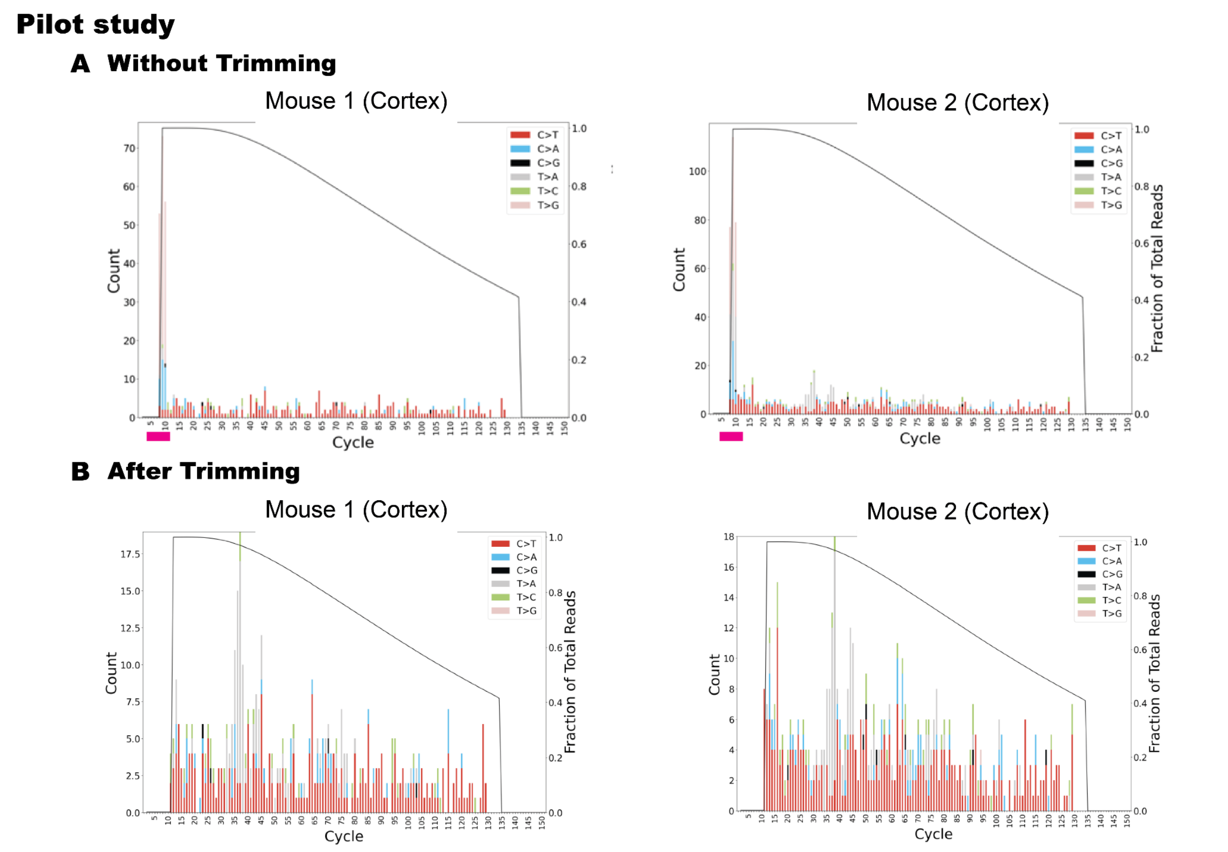

Fig. S1: Pilot study informed trimming of 5’ end of duplex reads by 10 bp in this study. (A) Mutation calls across the read before trimming. This data is for a pilot study where the mitochondrial genomes in the cerebellum and cortex of B6 mice were duplex sequenced. The 5’ end of the duplex read is highlighted in magenta. The x-axis denotes position along the read while the y-axis denotes counts of each mutation type: C>T (red), C>A (blue), C>G (black), T>A (lavender), T>C (green), T>G (pink). (B) The samples shown in (A) after trimming 10 bp from the 5’ end. Note that the y-axis scalings differ across panels.

Fig S2: Strand asymmetry varies across haplotypes, age, and tissues

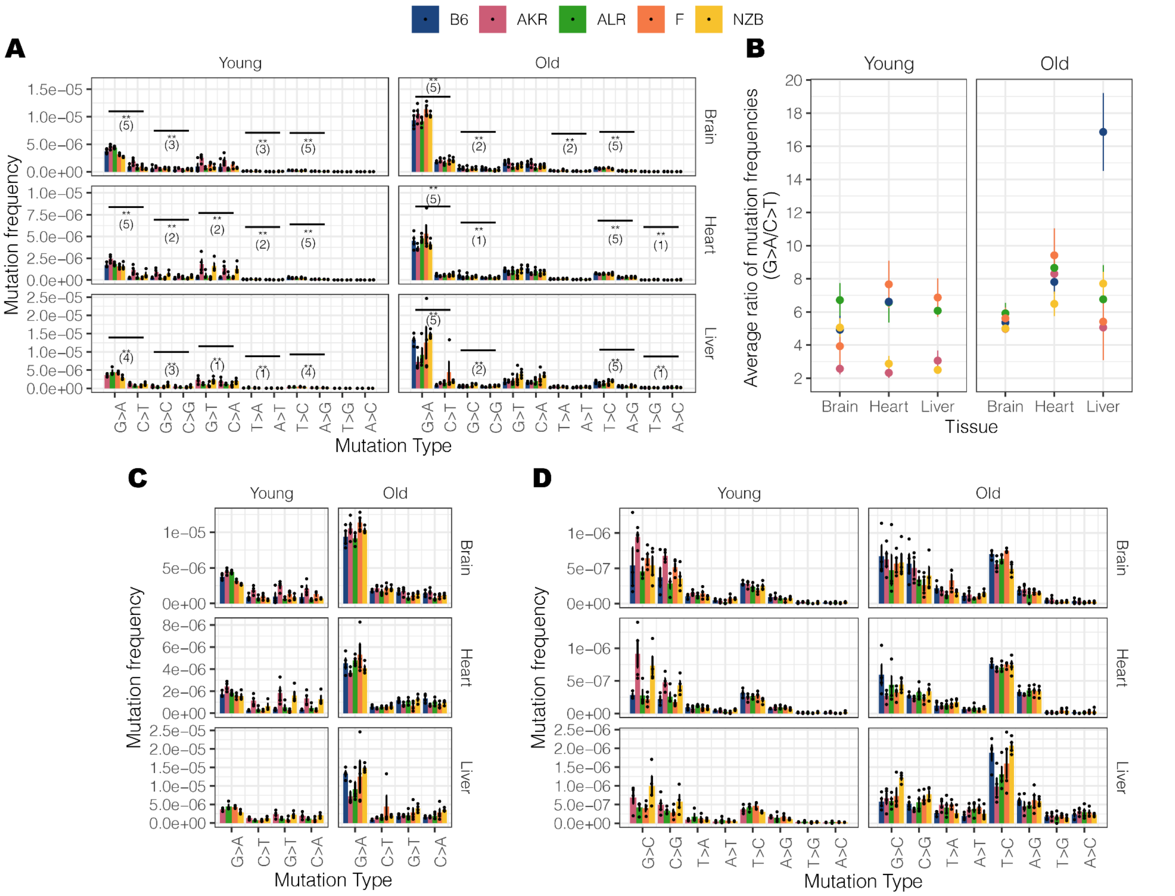

Fig. S2: Strand asymmetry varies across haplotypes, age, and tissues. (A) Mutation strand asymmetry is compared between the light strand (L-strand) and heavy strand. Mutations are called and referenced with respect to the L-strand. Mutation frequencies were calculated as the count of mutations of that type divided by the number of times the reference base was duplex sequenced. Error bars denote the standard error of the mean. Points represent the mutation frequency for each sample. Significance denotes strand bias for mutation types; adjusted p-value < 0.01 (two-sided Fisher's Exact test, Benjimi-Hochberg correction). The number of haplotypes with strand bias for respective mutation types is denoted in parentheses (maximum number = 5 mt-haplotypes). For exact p-values for each mutation pair comparison reference **Table S2.** (B) The strand asymmetry for G>A vs. C>T mutation types is compared across mt-haplotypes. The ratio of G>A to C>T mutation frequencies is averaged across samples within an experimental condition. Error bars denote the standard error of the mean. (C) Frequencies for G>A, C>T, G>T, and C>A mutation types. Error bars denote the standard error of the mean. Points represent the mutation frequency for each sample. (D) Frequencies for G>A, C>G, T>A, A>T, T>C, A>G, T>G, and A>C mutation types. Error bars denote the standard error of the mean. Points represent the mutation frequency for each sample. Note for **Fig S2C** and **Fig S2D** the y-axis scales differ between figures and tissues to capture smaller mutation frequencies. Mutations with an alternate allele depth > 100 and a mutation frequency > 1% were excluded. Each mutation is scored once to create a proxy mutation frequency for *de novo* mutations. The averages calculated for each analysis were across samples in each experimental condition: (n = 4 mice, except for B6-Young-Heart n = 3 mice).

Fig S3: Comparison of Sanchez-Contreras et al. 2023 and Serrano et al. 2023 unique mutation frequencies and strand asymmetry

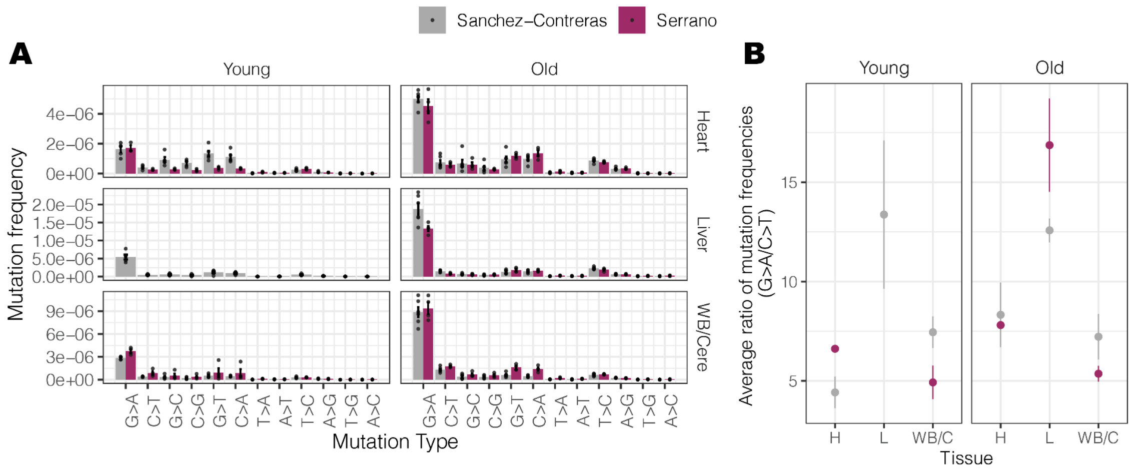

Fig. S3: Comparison of Sanchez Contreras et al. 2023 and Serrano et al. 2023 unique mutation frequencies and strand asymmetry. Mutation frequencies in B6 mice are featured, where the whole brain (Serrano et al.) is compared to cerebellum (Sanchez-Contreras et al.). Mutations with an alternate allele depth > 100 and a mutation frequency > 1% were excluded. Each mutation is scored once to create a proxy mutation frequency for *de novo* mutations as described in Sanchez-Contreras et al. 2023[1]. (A) Mutation frequencies were compared between the Sanchez-Contreras et al. and Serrano et al. studies. Mutation frequencies were calculated as the count of mutations of that type divided by the number of times the reference base was duplex sequenced. The average mutation frequency across samples in experimental conditions is plotted with error bars denoting the standard error of the mean. Points denote the mutation frequency for each sample. Corresponding statistical testing (two-sided t-test) is provided in **Table S3**. (B) The strand asymmetry for G>A and C>T mutations is compared across studies. The ratio of G>A to C>T mutation frequencies was calculated. The error bars denote the standard error of the mean. Mice that were chemically treated in the Sanchez-Contreras et al. study were excluded from these analyses as chemical treatments were shown to modulate the mutational landscape. The averages calculated for each analysis were across samples in each experimental condition (young n = 5 mice Sanchez-Contreras et al. 2023; aged n = 6 mice Sanchez-Contreras et al. 2023; n = 4 mice Serrano et al., except B6-Young-Heart n = 3 mice).

Fig S4: Mitochondrial Copy Number

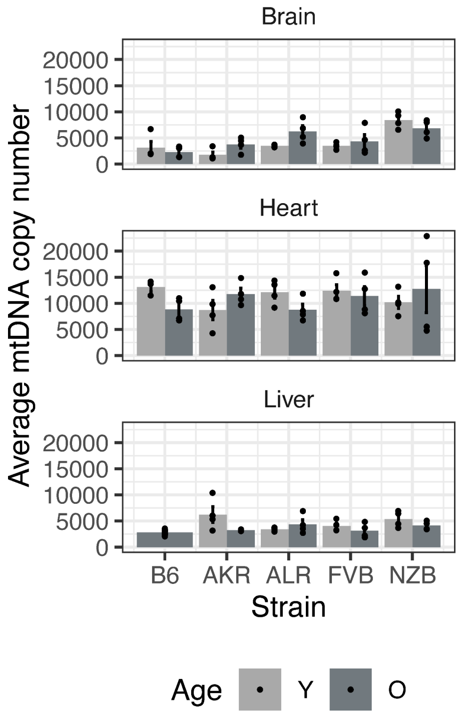

Fig. S4: Mitochondrial copy number. Mitochondrial and nuclear copy numbers were quantified using qPCR. The mitochondrial to nuclear copy number ratio is used as an estimate of mitochondrial copy number in a tissue. The average mitochondrial DNA copy number across samples in an experimental condition for young (light gray) and aged (dark gray) mice is featured with error bars denoting the standard error of the mean (n = 4 mice, except for B6, Young, Heart n = 3 mice).

Fig S5: Proportion of alleles in the tRNA^{Arg} 8 base mutation peak region

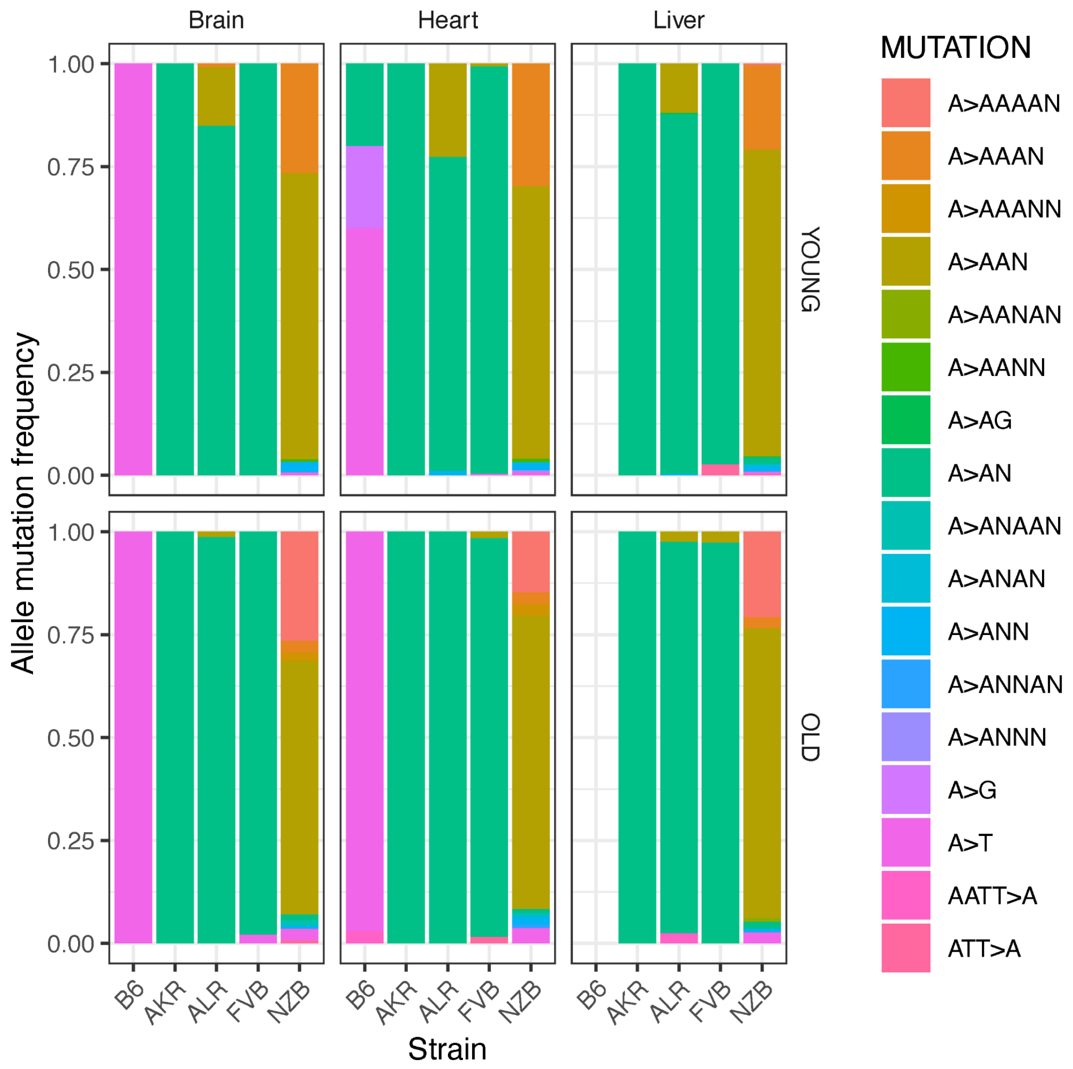

Fig. S5: Proportion of alleles in the tRNA^{Arg} 8 base mutation peak region. The proportion of each allele present in the A repeat region of the DHU arm of *mt-tRNA^{Arg}* was calculated as the total number of each mutation type divided by the total number of mutations that occur in this region. This proportion was calculated for each experimental condition. Mutations that occur at a frequency > $1x10^{-3}$ were excluded from the analysis, including the previously known heteroplasmic frequency in *mt-tRNA^{Arg}* across conplastic strains[2]. The mutation key (REF>ALT) denotes the mutation that occurs in *mt-tRNA^{Arg}*, Ns in the ALT allele represent bases where a duplex consensus could not be reached.

Fig S6: Computationally predicted changes in $tRNA^{Arg}$ structures

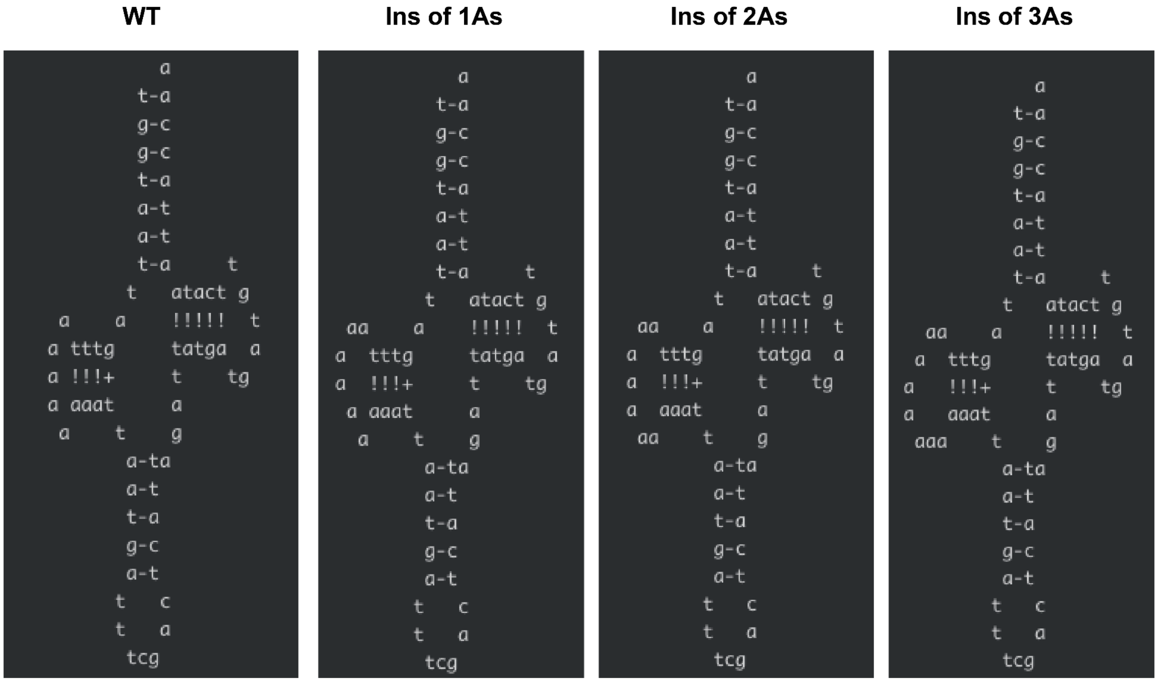

Fig. S6: Computationally predicted changes in $tRNA^{Arg}$ structures. Structures were created using ARWEN (v1.2) [3]. Structures were created for the 3 most abundant mutation types across conditions **(Fig. S5)**: 1-3 A insertion in the DHU A-repeat region. As were inserted at position 9819 since we lack the resolution to locate the exact position an A-insertion occurs.

Fig S7: Change in SNVs, deletions, and insertions with age across all tissues

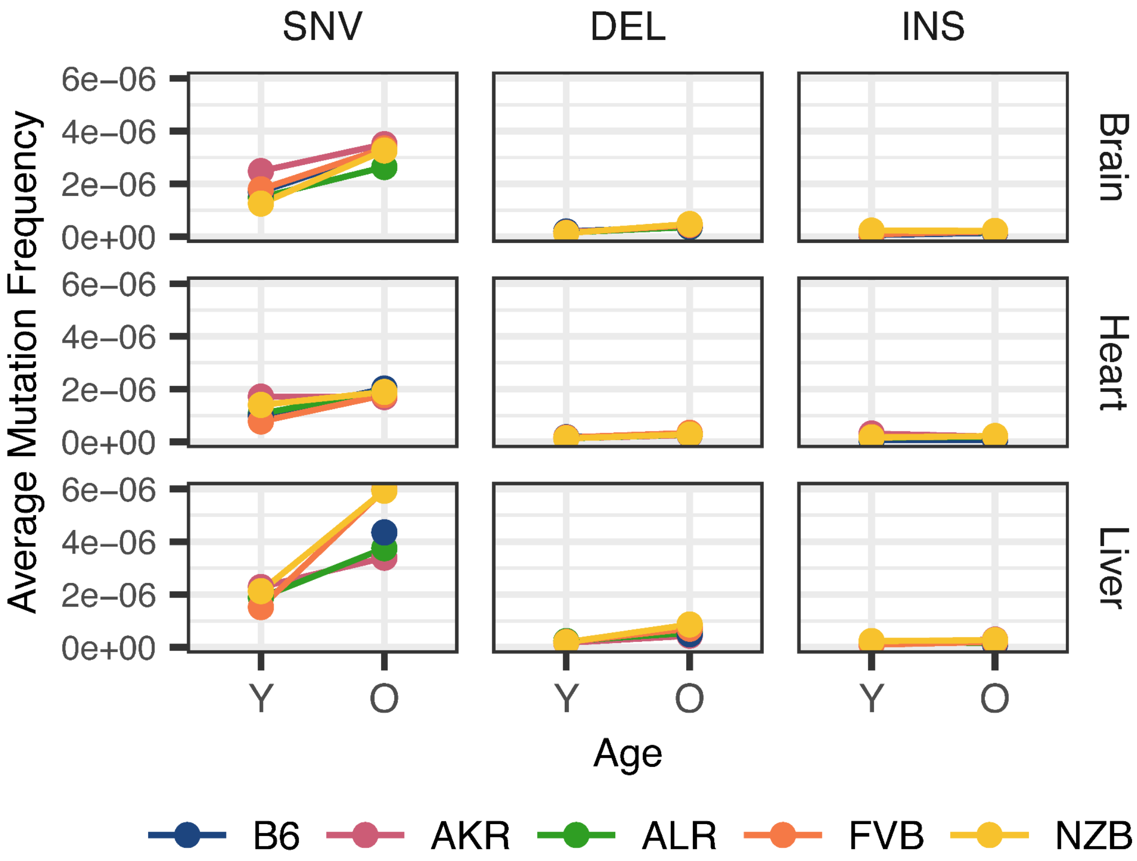

Fig. S7: Change in SNVs, deletions, and insertions with age across all tissues. The average mutation frequency is compared between mt-haplotypes for different classes of mutations: single nucleotide variants (SNVs), deletions, and insertions. The average mutation frequency was calculated as the sum of alternative alleles in a class divided by the total duplex bp depth. Mutations with a frequency > $1x10^{-3}$ were excluded from this analysis. Mutation counts and duplex depth were aggregated across samples in experimental conditions (n = 29 conditions; n = 4 mice, except for B6-Young-Heart n = 3 mice).

Fig S8: Estimation of the optimal number of mutational signatures

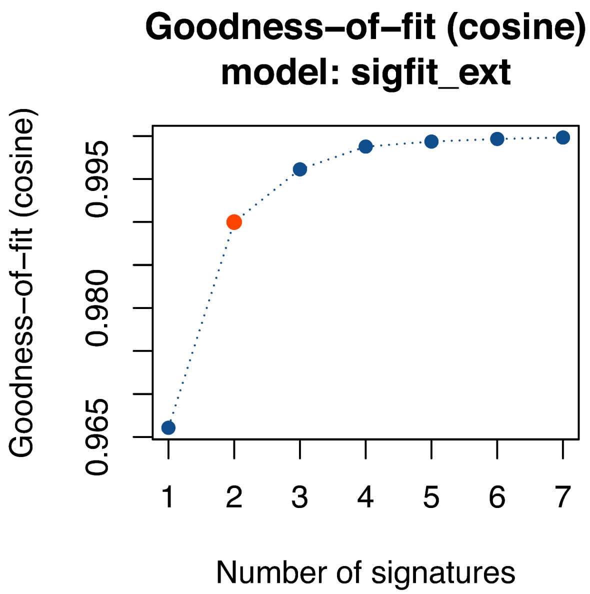

Fig. S8: Estimation of the optimal number of mutational signatures. Observed counts for each mutation type were used for signature extraction using sigfit. A range of signatures (1-7) was given for possible signature extraction. Sigfit highlights the best number of signatures (as shown in red; signatures = 2), based on a cosine similarity metric that compares the original catalog of mutational signatures and the sigfit inferred signatures, as previously described in [4].

Fig S9: Mutation frequencies for macaque and mouse tissues.

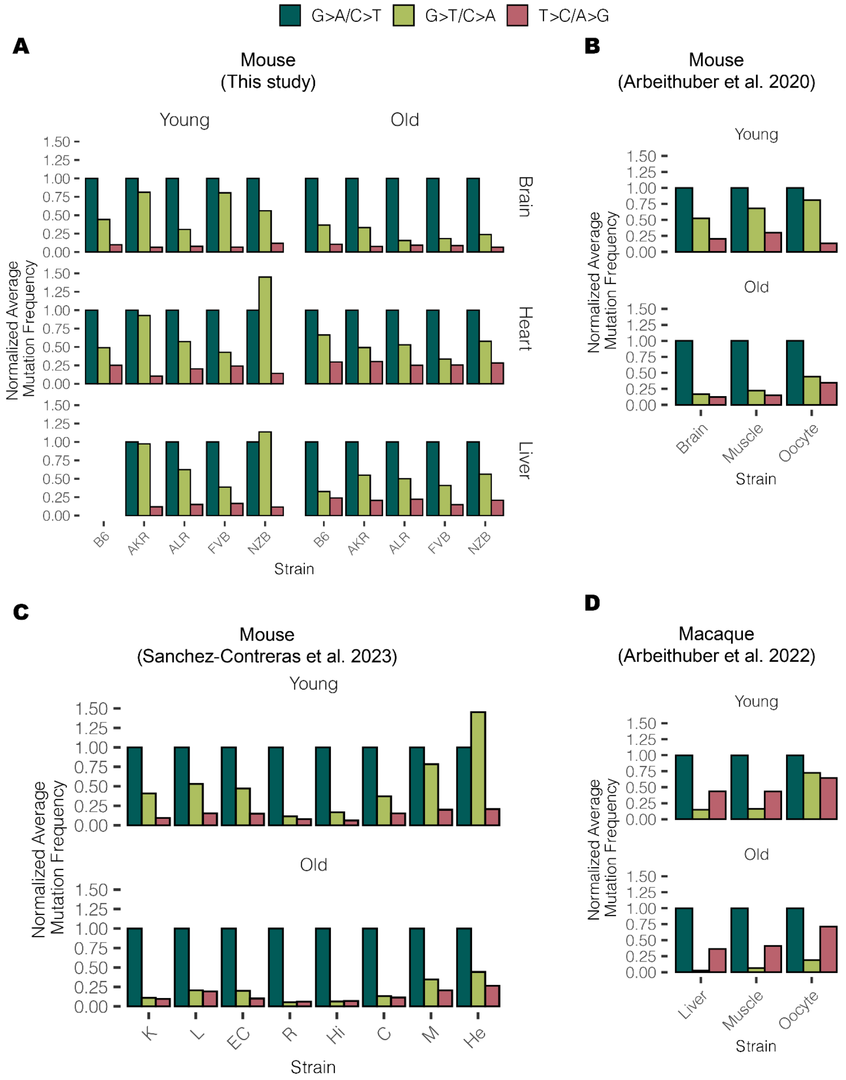

Fig. S9: Mutation frequencies for macaque and mouse tissues. The mutation frequency for *de novo point* mutations was calculated as the total count of mutations of the given type divided by the duplex bp depth for the reference nucleotide. Counts and duplex depth for complementary mutation types were aggregated. These mutation frequencies were then normalized to the G>A/C>T frequency, which is consistently the most abundant mutation type. (A) The normalized average mutation frequency for the three most abundant mutations in this study. Counts and depths across samples in an experimental condition were aggregatedm (n = 29 conditions, n = 4 mice per condition except B6-Young-Heart n = 3 mice). (B) The normalized mutation frequency across three mouse tissues in Arbeithuber et al. 2020[5] (n = 28 mice for young; n = 8 mice for old). (C) The normalized mutation frequency across mouse tissues in Sanchez-Contreras et al. 2023[1] (n = 5 mice for young; n = 6 mice for old). Tissue labels are as follows: kidney (K), liver (L), RPE/choroid (RC), retina (R), hippocampus (Hi), cerebellum (C), skeletal muscle (M), and heart (He). (D) The normalized mutation frequency across macaque tissues in Arbeithuber el al. 2022 [6] (n = 9 macaques for young, n = 7 macaques for old).

Fig S10: Position of observed $\frac{hN}{hS}$ statistics in the distribution of simulated $\frac{hN}{hS}$ statistics

Frequency Bins (1e−2, 1] (1e−3, 1e−2] (1e−4, 1e−3] (5e−5, 1e−4] (0, 5e−5]

Condition: Young Brain

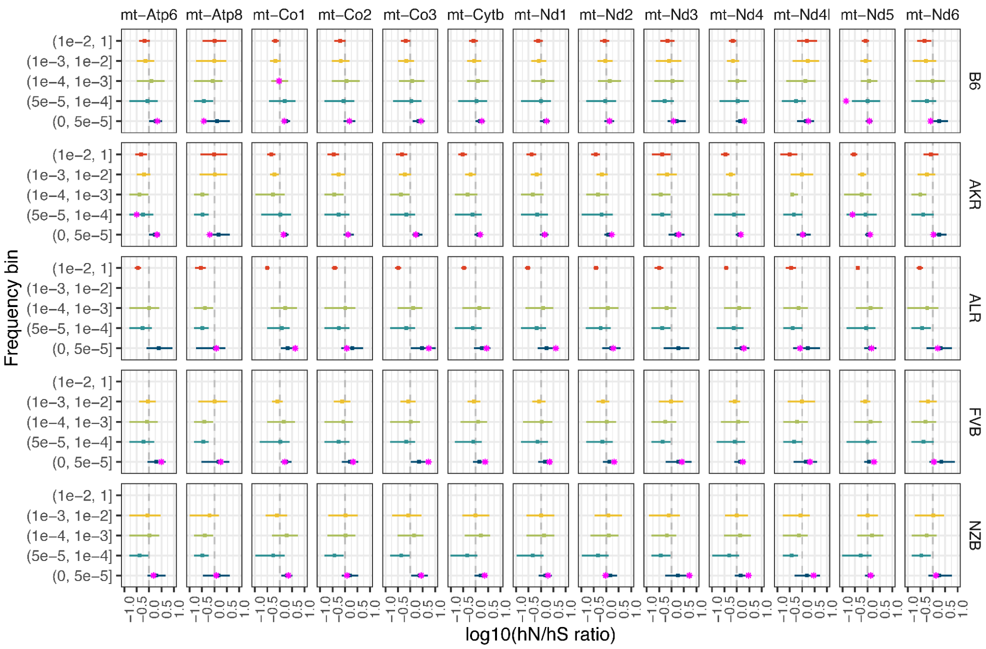

Condition: Old Brain

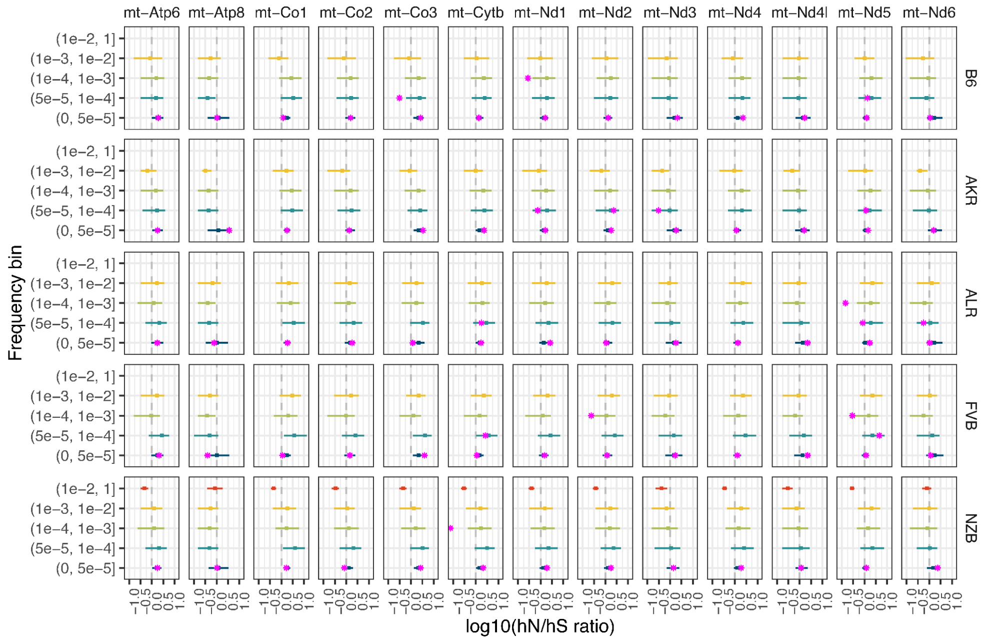

Condition: Young Heart

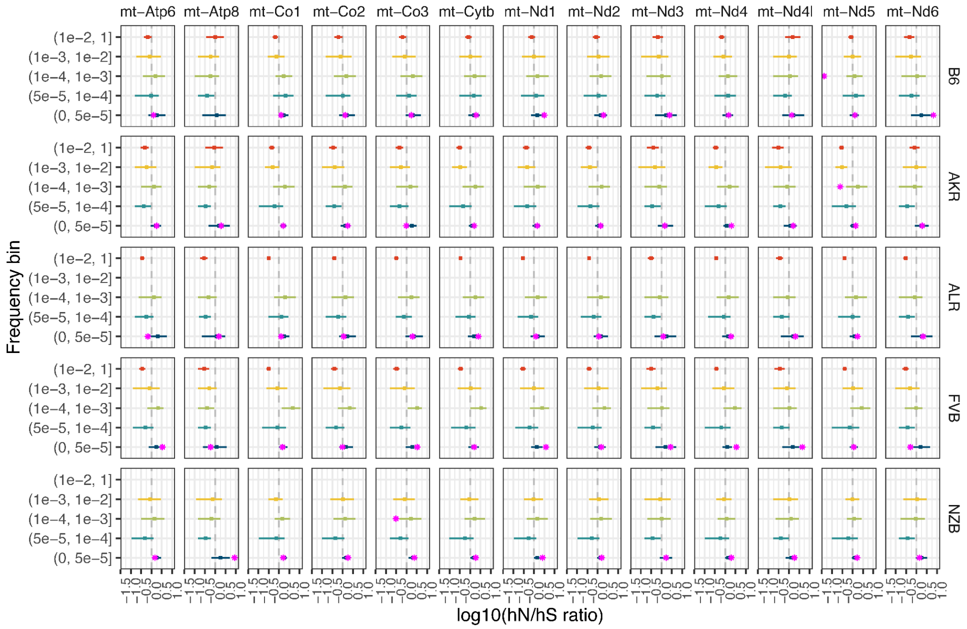

Condition: Old Heart

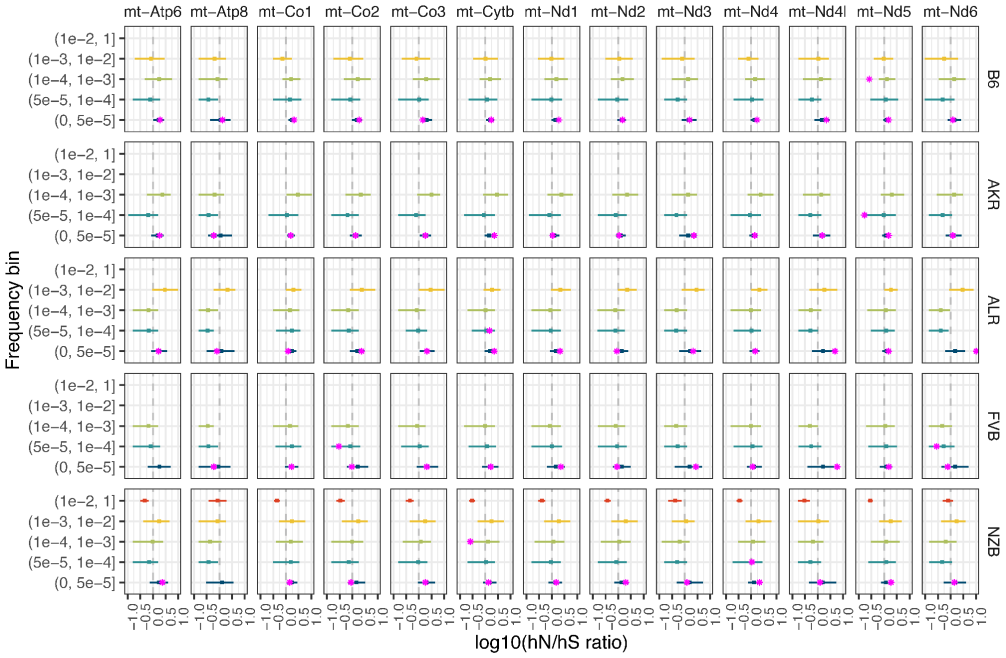

Condition: Young Liver

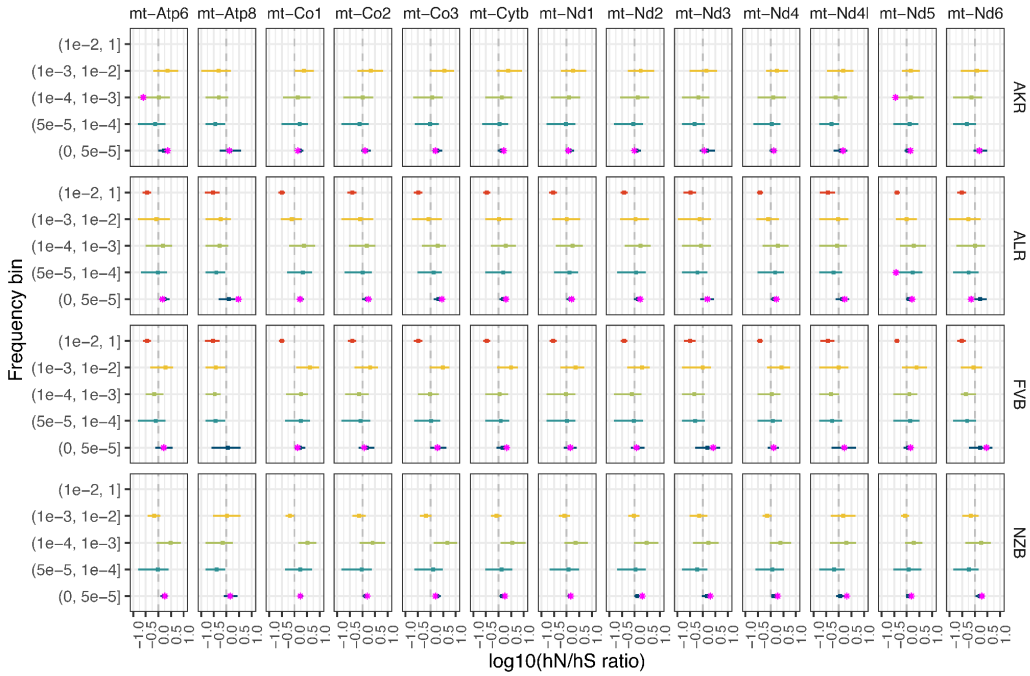

Condition: Old Liver

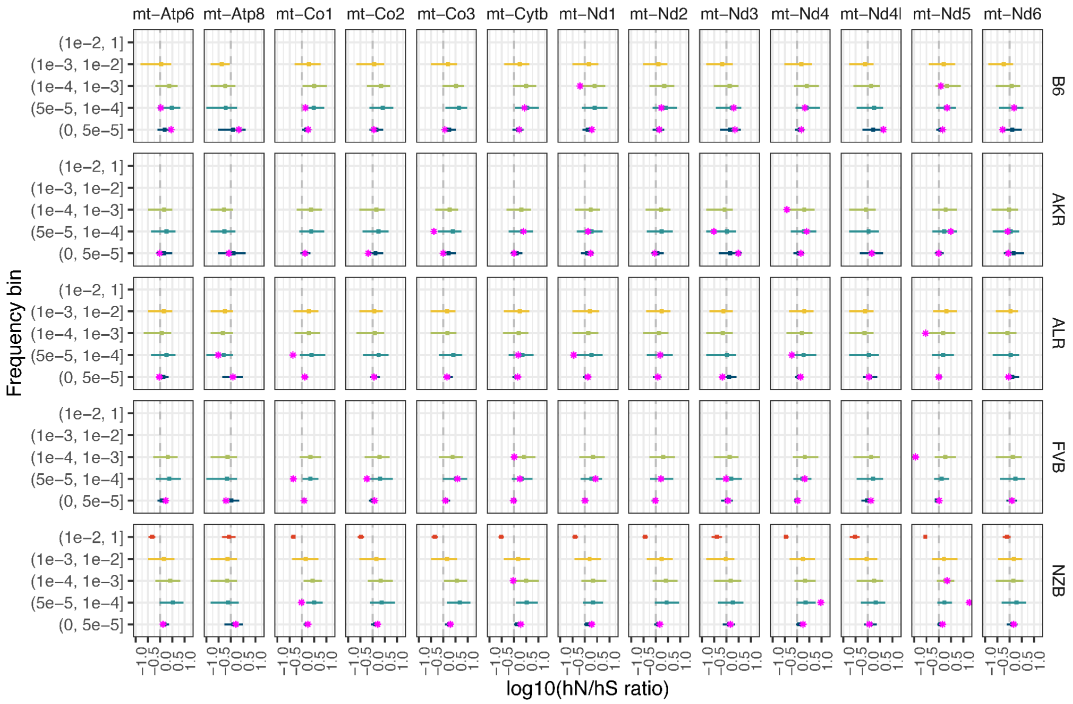

Simulated $\frac{hN}{hS}$ statistics were generated as described in Methods: Testing for selection. Shown are the average simulated $\frac{hN}{hS}$ statistics. The segment denotes the range in simulated ratios extending from the 2.5 percent quantile to the 97.5 percent quantile. The $\frac{hN}{hS}$ statistics are in log10 scale, where the dotted, gray vertical line denotes theoretical neutrality. The magenta asterisk indicates the observed $\frac{hN}{hS}$ statistic for each bin. There are cases where either the simulated and/or observed $\frac{hN}{hS}$ statistics could not be calculated:

- Absent observed statistic, but simulated distribution generated: either hN or hS equals 0. Due to this, the observed ratio was excluded from this study. Note, the observed mutation proportions and counts could generate nonzero hN and hS values for a gene in a simulation.
- Absent simulated distribution and observed ratio: there are zero mutations in the given mutation bin for the experimental condition

10,000 simulations were performed for each *frequency bin x strain x age x tissue* combination. Simulated ratios were filtered as explained above. The mutation counts were combined across samples in experimental conditions to generate the observed mutation spectra and observed $\frac{hN}{hS}$ statistics (n = 29 conditions; n = 4 mice, except B6-Young-Heart n = 3 mice).

Fig S11: Mutational hotspots in Sanchez-Contreras et. al (2023)

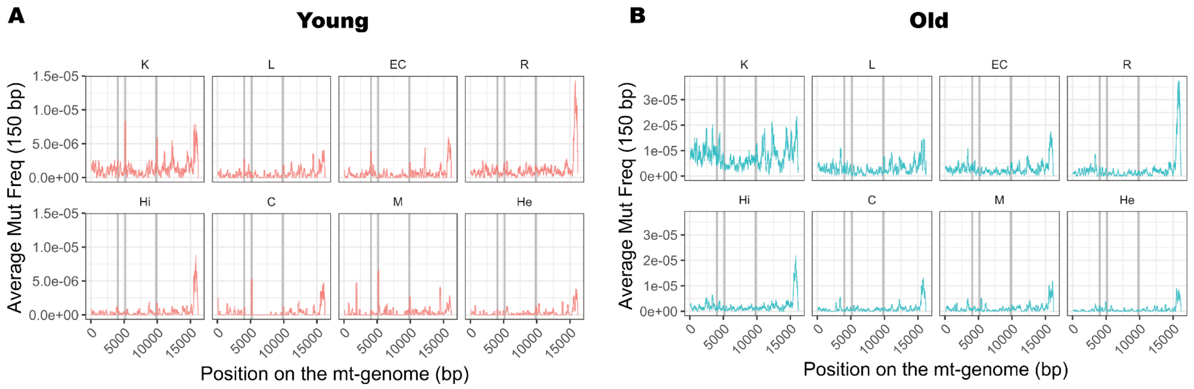

Fig. S11: Mutational hotspots in Sanchez-Contreras et al. (2023). In Sanchez-Contreras et al. (2023), B6 mice were used. (A) The average mutation frequency in a 150 bp sliding window is shown for young mice. (B) The average mutation frequency in a 150 bp sliding window is shown for aged mice. Mutation counts and depths for replicates in an experimental condition were aggregated. The gray bands denote where the *OriL* (pos 5159-5190), *mt-Nd2* (pos 4030-4070), and *mt*-t*RNA*^{Arg} (pos 9807-9874) are located. For *mt-Nd2*, the region containing the mutational hotspot in this study is highlighted. Tissue labels are as follows: kidney (K), liver (L), RPE/choroid (RC), retina (R), hippocampus (Hi), cerebellum (C), skeletal muscle (M), and heart (He).

Fig S12: Validation of conplastic strains

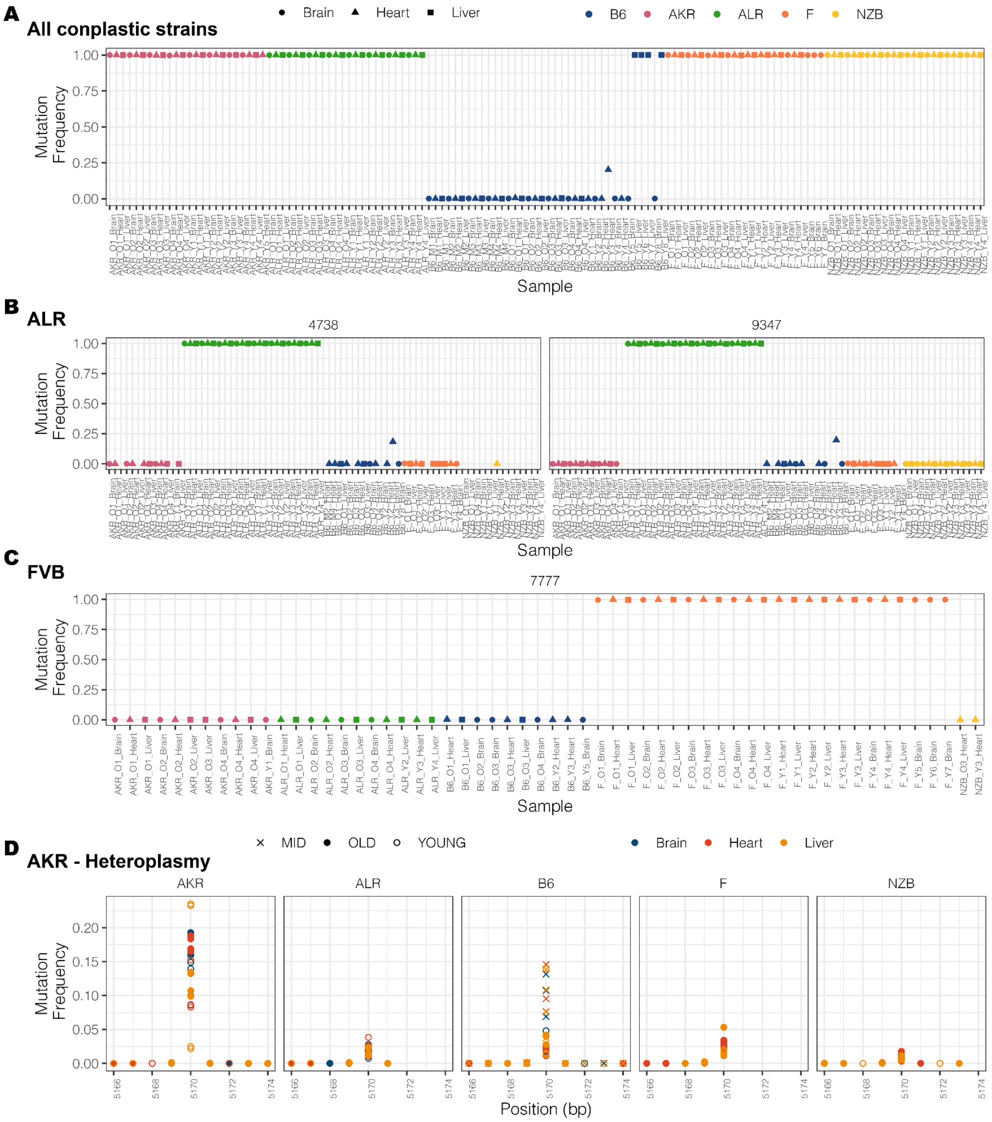

Fig. S12: Validation of conplastic strains. The mutation frequency, calculated as the number of alternative alleles divided by the number of duplex mt-genomes at the position, was checked for each haplotype site. (A) The mutation frequency for the T>C mutation at position 9460 in the gene *mt-ND3.* All conplastic strains differ from B6 (blue) at this position. B6 young liver samples that had this haplotype present were excluded from the study due to potential contamination. (B) The mutation frequency for ALR (green) haplotype sites positions 4738 (*mt-ND2*) and 9347 (*mt-COXIII)*. The B6 young heart sample with presence of this haplotype was excluded from the study due to potential contamination. (C) The mutation frequency for the FVB (orange) haplotype site at position 7777 (*mt-Atp8)*. For (A) - (C), color denotes strain (B6 (blue), AKR (pink), ALR (green), FVB (orange), NZB (yellow)) and shape denotes tissue (brain (circle), heart (triangle), liver (square)). (D) Comparison of the heteroplasmic allele frequency in the *OriL*. Shape denotes age (mid (cross), old (filled circle), young (hollow circle)) and color denotes tissue (brain (blue), heart (red), liver (yellow)).

Fig S13 Trinucleotide Spectra

The trinucleotide spectra for all experimental conditions is depicted below. Only *de novo mutations* were used in this analysis and were identified by filtering mutations with 1) an alternative allele depth < 100 and 2) a mutation frequency < 0.01. Each mutation is scored once to create a proxy mutation frequency for *de novo* mutations. The mutation fraction is the proportion of each mutation type in a given trinucleotide context divided by the total count of *de novo* mutations for a condition. The trinucleotide and mutation type featured represent mutations on either strand. For example, ACG represents both ACG and CGT, where either a C>T or a G>A mutation has occurred.

Young Brain

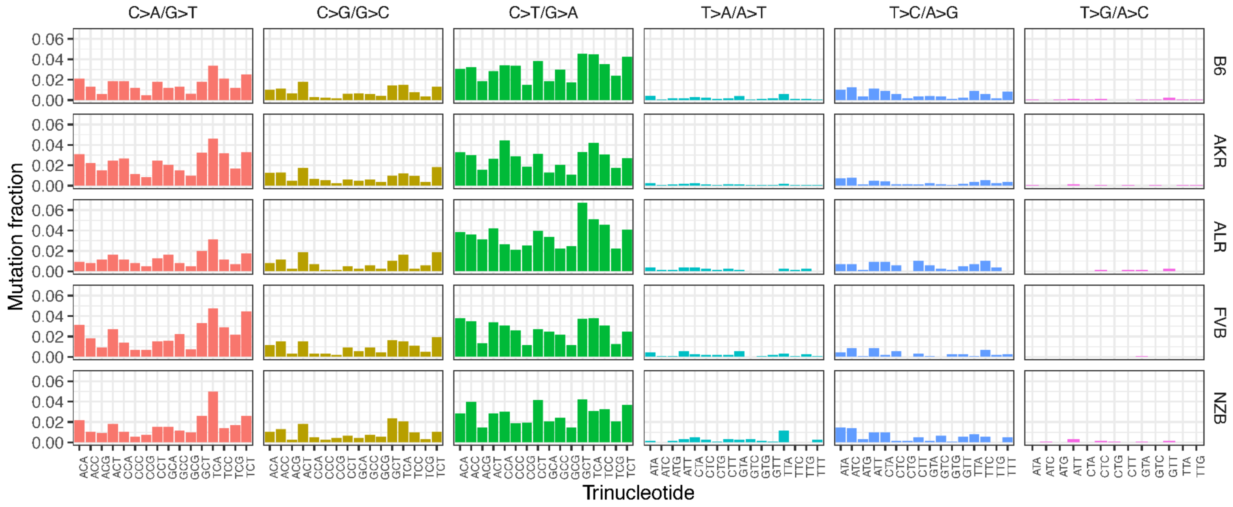

Old Brain

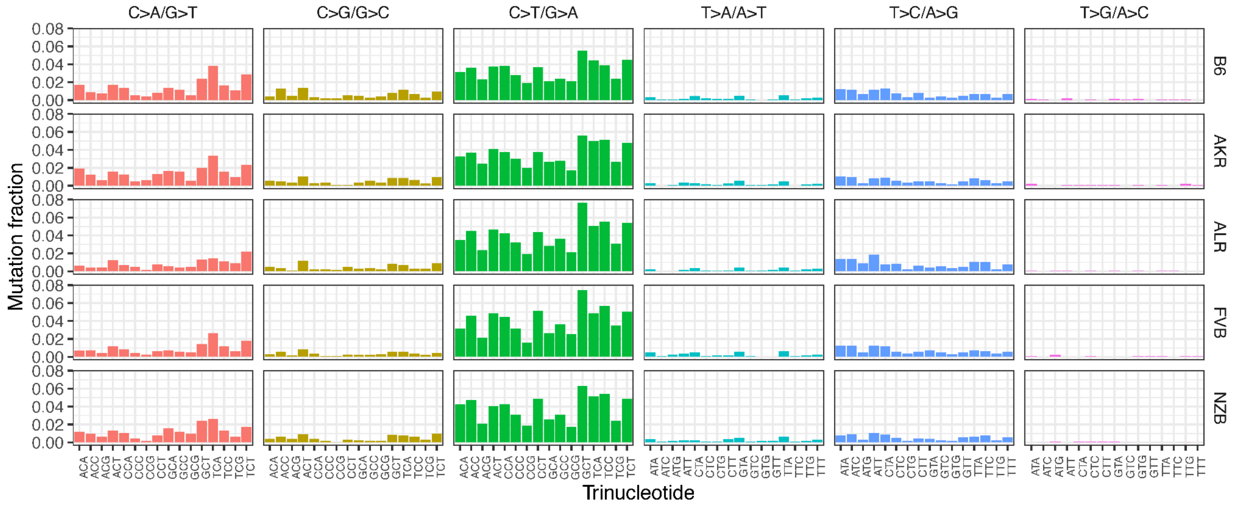

Young Heart

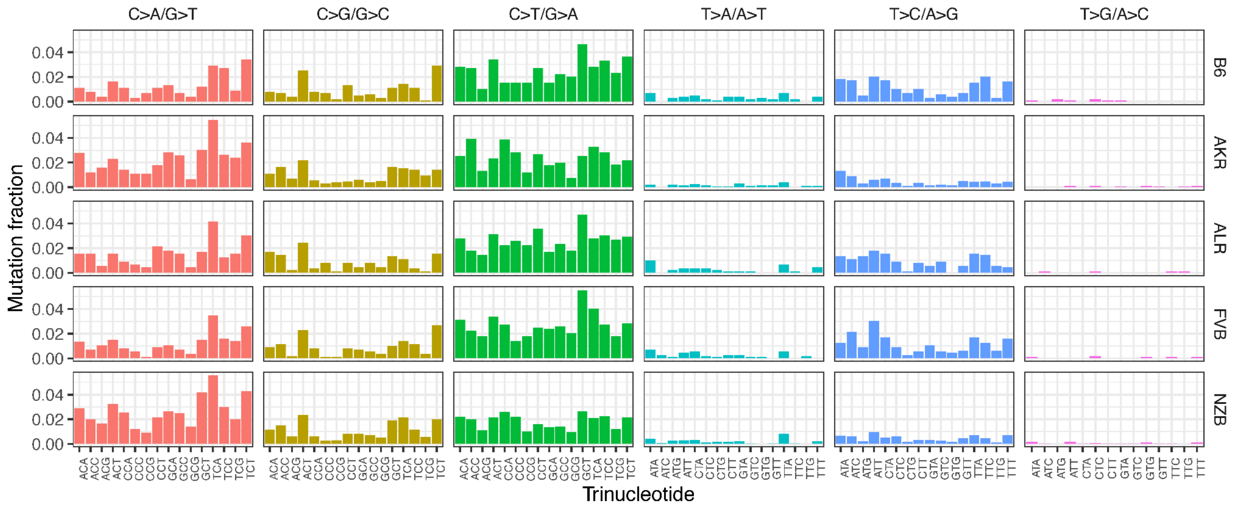

Old Heart

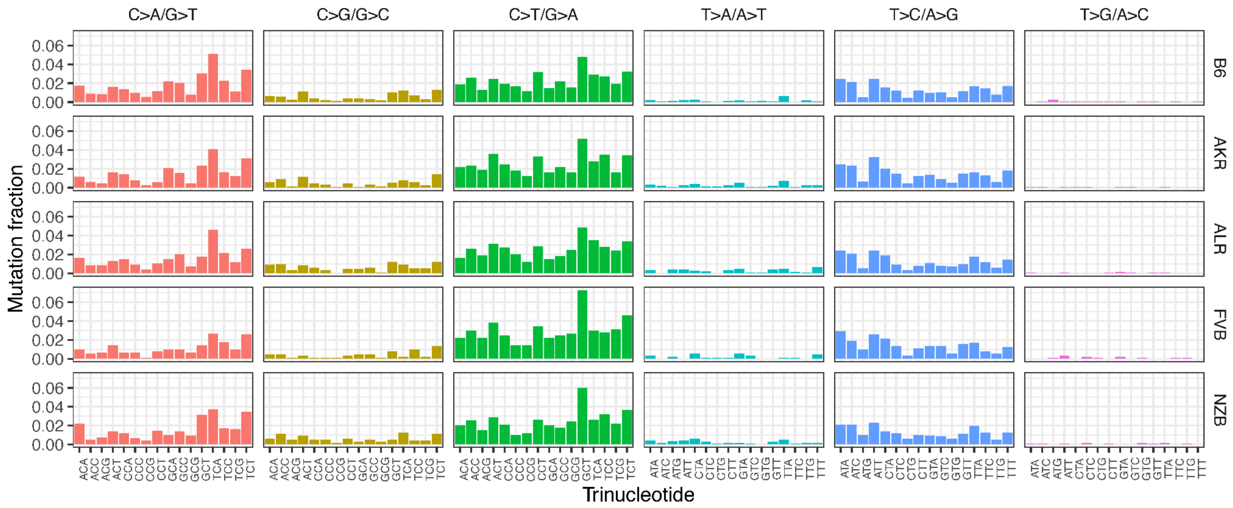

Young Liver

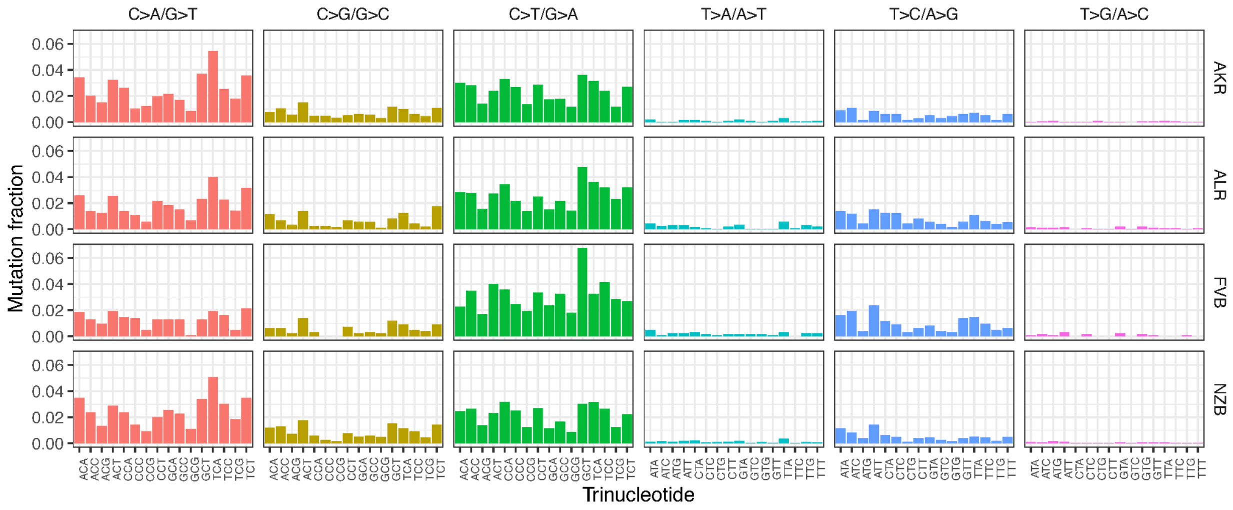

Old Liver

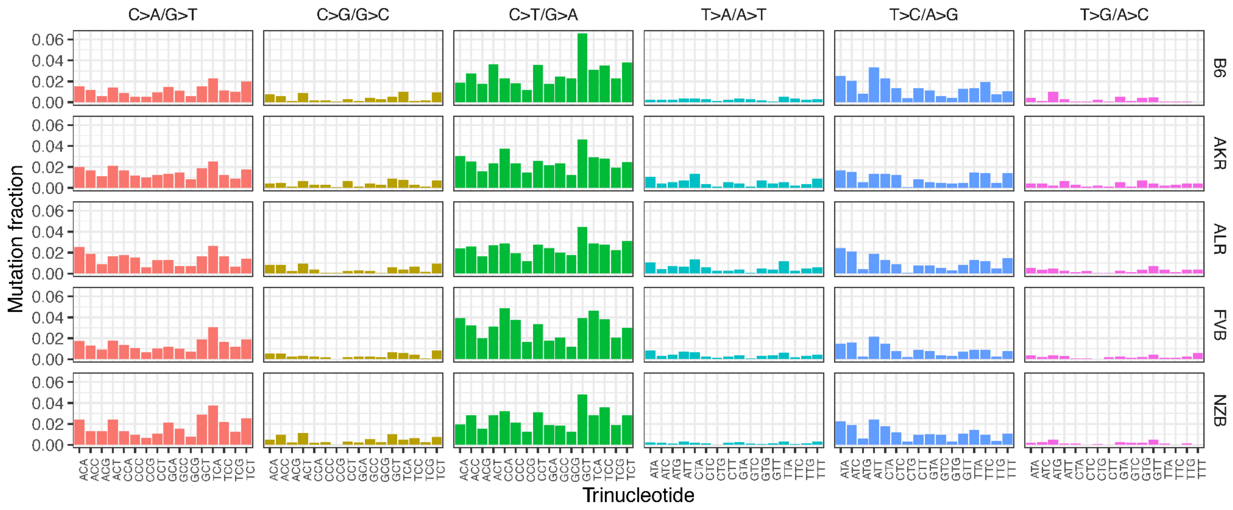

Fig S14: Distribution of mutation counts and proportion of genes under selection across frequency bins

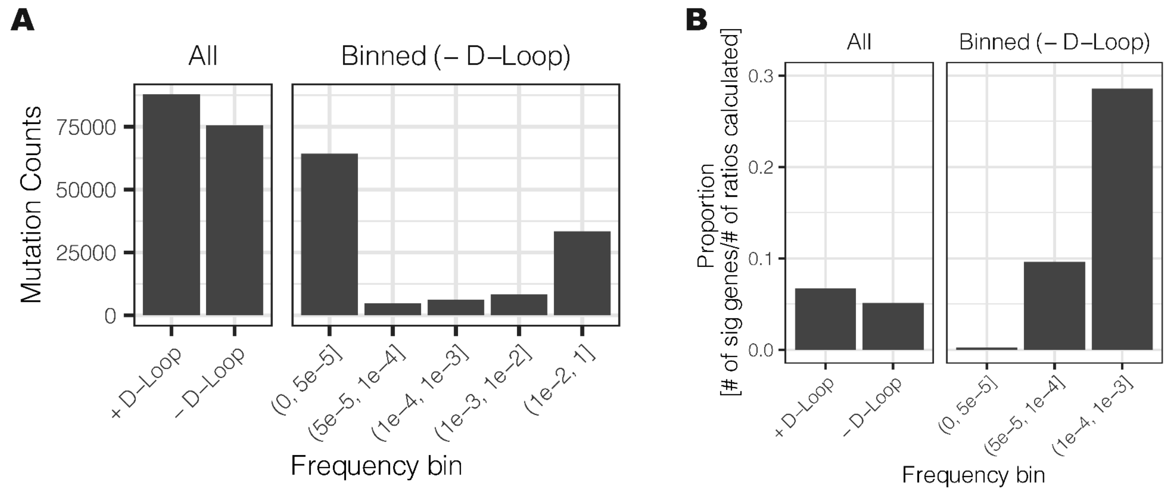

Fig. S14: Distribution of mutation counts and proportion of genes under selection across frequency bins. (A) Count of total mutations in each analysis and frequency bin. Mutation counts were calculated as the sum of the duplex alternative allele depth across experimental conditions. For analyses labeled "all", mutation counts were aggregated regardless of frequency. These mutation counts were used to simulate $\frac{hN}{hS}$ statistics. (B) The proportion of genes under selection in each analysis. Within each bin and the aggregated analyses, the proportion is calculated as the count of genes under selection divided by the count of observed $\frac{hN}{hS}$ statistics that could be calculated. Note: $\frac{hN}{hS}$ statistics were excluded from the study if *hN* or *hS* equaled 0. For the aggregated analyses (labeled "All"), mutations with a frequency greater than $1x10^{-3}$ were excluded. For the binned frequencies, mutations in the D-Loop were excluded.

Fig S15: Mutational spectra for nonsynonymous and synonymous variants

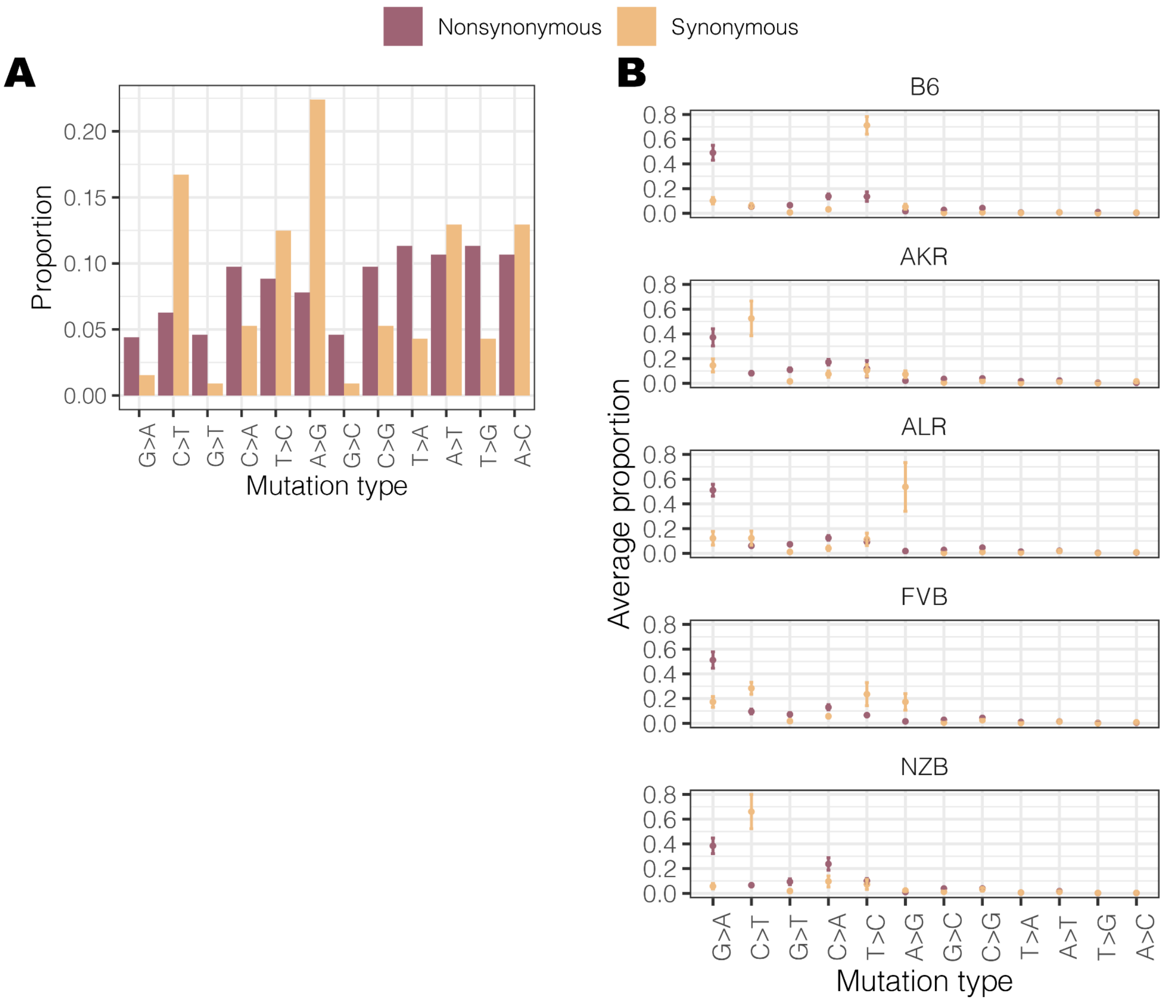

Fig. S15: Mutational spectra for nonsynonymous and synonymous mutations. (A) The null mutational spectra for synonymous (orange) and nonsynonymous (purple) mutations across experimental conditions. For every mutation type, the number of nonsynonymous and synonymous mutations a given mutation type can produce was calculated. The proportion was calculated as the count of nonsynonymous or synonymous mutations resulting from a given mutation type divided by all possible nonsynonymous or synonymous mutations in the mt-genome. (B) The proportion of each mutation type comprising the total count of nonsynonymous (purple) and synonymous (orange) mutations. The average proportion across *tissues x age* (n = 6 conditions, except for B6 where n = 5 conditions) for each mt-haplotype is shown with error bars denoting the standard error of the mean.

Fig S16: Estimation of NUMT contamination

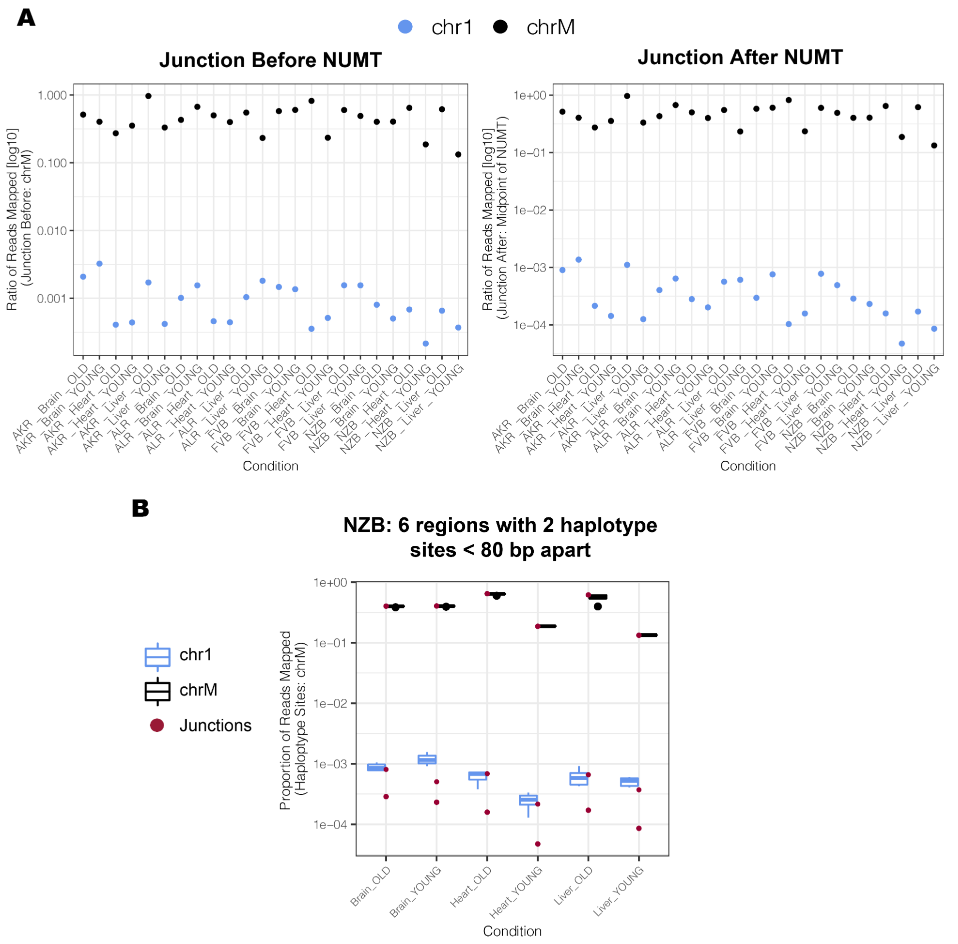

Fig. S16: Estimation of NUMT contamination. (A) To estimate for NUMT contamination paired end duplex sequencing reads were remapped to mm10 with an unmasked chr1 NUMT. The number of reads that map to 10 bp upstream and 10 bp downstream of the NUMT region, referred to as the junction regions, was calculated. The figures denote the number of duplex reads that map to the junction regions divided by the average duplex depth of the corresponding region in the mt-genome with the chr1 NUMT masked (blue). Shown is also the number of reads that map to the corresponding junction regions in the mt-genome divided by the average duplex depth of the corresponding region in the mt-genome with the chr1 NUMT masked (black). (B) Duplex sequencing reads for the NZB strain were remapped using a NZB reference mt-genome. Reads were aggregated across samples in each experimental condition (n = 4 mice per experimental condition *strain x age*). Six clusters of haplotype sites (regions where more than one haplotype site exists in a read) were identified. The distribution of the proportion of reads mapping the chr1 and those mapping to the mt-genome are depicted in boxplots. The center line in the boxplot highlights the median and the bounds of the box represent the first and third quartiles. The whiskers depict the maximum and minimum values defined by the sum of the third quartile and 1.5 multiplied by the interquartile range and the difference between the first quartile and 1.5 multiplied by the interquartile range, respectively.". The proportion of reads mapping to chr1 and the mt-genome at the junction sites (shown in A) are represented in red.

1. Sanchez-Contreras, M. *et al.* The multi-tissue landscape of somatic mtdna mutations indicates tissue specific accumulation and removal in aging. *Elife* **12**, e83395 (2023).
2. Yu, X. *et al.* Dissecting the effects of mtDNA variations on complex traits using mouse conplastic strains. *Genome Res.* **19**, 159–165 (2009).
3. Laslett, D. & Canbäck, B. ARWEN: a program to detect tRNA genes in metazoan mitochondrial nucleotide sequences. *Bioinformatics* **24**, 172–175 (2008).
4. Gori, K. & Baez-Ortega, A. sigfit: flexible Bayesian inference of mutational signatures. *bioRxiv* 372896 (2020) doi:10.1101/372896.
5. Arbeithuber, B. *et al.* Age-related accumulation of de novo mitochondrial mutations in mammalian oocytes and somatic tissues. *PLoS Biol.* **18**, e3000745 (2020).
6. Arbeithuber, B. *et al.* Advanced age increases frequencies of de novo mitochondrial mutations in macaque oocytes and somatic tissues. *Proc. Natl. Acad. Sci. U. S. A.* **119**, e2118740119 (2022).
